# Editorial: Complex Post-Traumatic Stress Disorder in the Context of Human Rights Abuse

**DOI:** 10.3389/fpsyt.2017.00142

**Published:** 2017-08-14

**Authors:** Cornelius Katona, Katy Robjant

**Affiliations:** ^1^Helen Bamber Foundation, London, United Kingdom

**Keywords:** complex PTSD, narrative exposure therapy, conflict, human trafficking, integrated care

**Editorial on the Research Topic**

**Complex Post-Traumatic Stress Disorder in the Context of Human Rights Abuse**

## Introduction

The papers in this small volume all address the needs, clinical presentations, and possible treatment approaches for people who have suffered multiple and repeated trauma and who either remain in or have fled from conflict and/or abuse and exploitation.

## Needs Assessment

A thorough needs assessment is crucial to avoid the trap of focusing on the needs which are obvious to the assessing clinician(s) but may be of relatively minor importance to the traumatized individual. Such an approach itself has the potential to decrease the sense of helplessness and lack of self-worth that is a core feature of “complex PTSD” and provides a pathway to enabling traumatized individuals to play an active role in their treatment and rehabilitation. The work of Hecker et al. provides a good example of this in their description of the “appetitive aggression” that is often a key disabling feature in the presentation of former combatants and, if not addressed directly, provides a major barrier to their social reintegration.

## Complexity of Trauma and of Responses to Trauma

The importance of multiplicity of trauma increasingly recognized in the research literature, as reflected in all the papers in this volume. The dominant current approach invokes the concept of “complex PTSD,” which is likely to be incorporated in the forthcoming 11th edition of the International Classification of Diseases. In our view, this is a very welcome development, since the disturbances in self-regulation that form part of the “complex PTSD” definition may be profoundly disabling in their own right (1). As Silove et al. argue, however, PTSD and complex PTSD symptoms may best be conceptualized as components of a unitary response to extensive persecution and conflict-related trauma.

In this context, a systematic approach to diagnosis and the rating of symptom severity is important if new approaches are to be evaluated rigorously. Clinical assessments need also to be valid and culturally appropriate. Winkler et al. provide an example of good practice in that their measures of both depression and PTSD use local assessors trained in diagnostic interviewing in the local languages, as well as using locally defined cut-off points for rating scales widely used in international research in this field.

## Need for Locally Feasible and Evidence-Based Solutions

Two of the papers in this volume—Hecker et al. and Robjant et al.—present adaptations of an established evidence-based trauma-focused treatment, narrative exposure therapy [NET; (2)] for client groups with specific needs over and above their trauma symptoms. Hecker et al. outline a new approach for the treatment of former combatants and violent offenders, in which a key aim is to reduce risk of future violence at the same time as addressing PTSD symptoms. Robjant et al. provide a preliminary evaluation of NET in survivors of human trafficking, in which there is an additional emphasis on reducing the risk of re-trafficking through the avoidance of high-risk relationships. Although this study was only a retrospective audit, it does provide support for the feasibility and effectiveness of NET in a population in which it has not previously been evaluated.

## Conclusion

In our view, successful clinical management of such traumatized individuals requires a comprehensive assessment of clinical presentation (which often goes beyond conventional clinical descriptions of PTSD) and of their many and complex needs. Therapeutic approaches need to be feasible within the local context and culturally acceptable as well as being supported by the available evidence as to their clinical trial efficacy and real-world effectiveness. The impact of treatments needs to be evaluated in terms of functional improvement and reduction in vulnerability to future trauma as well as through change in trauma and related mental symptoms.

Individuals who have experienced multiple trauma are by definition likely to have many unmet needs. Treatments for PTSD, aggression, high-risk behaviors, and other trauma-related problems, are more likely to be effective if they are incorporated into an integrated model of care, in which an individual’s multiple needs (including legal protection, health, social, and practical needs) are met together as a whole, since as needs arise in any one of these areas, there is often a de-stabilizing effect in other areas. The papers in this short volume also highlight that where assistance is focused on the provision of treatment of trauma-related problems, NET offers an evidence-based solution (for further information please visit http://www.vivo.org).

## Author Contributions

CK and KR contributed equally.

## Conflict of Interest Statement

The authors declare that the research was conducted in the absence of any commercial or financial relationships that could be construed as a potential conflict of interest.
